# Correction: Soil Bacterial Communities Respond to Mowing and Nutrient Addition in a Steppe Ecosystem

**DOI:** 10.1371/journal.pone.0089711

**Published:** 2014-02-26

**Authors:** The PLOS ONE Staff

The Funding statement is incorrect. The Funding statement should read: “This work was supported by the National Natural Science Foundation (31300431, 31170433) and State Key Laboratory of Forest and Soil Ecology (LFSE2013-15) of China.”
